# Digital Health Intervention for and Long-Term Health Outcomes of a Divorce Cohort With Linked Danish Data: 5-Year Posttrial Follow-Up of a Randomized Controlled Trial

**DOI:** 10.2196/69387

**Published:** 2025-12-30

**Authors:** Andreas Nielsen Hald, Peter Fallesen, Frank Eriksson, Gert Martin Hald

**Affiliations:** 1 Section of Health Services Research, Department of Public Health, Aarhus University, Aarhus, Central Jutland, Denmark; 2 Swedish Institute for Social Research, Stockholm University, Stockholm, 106 91, Sweden, 46 08 16 20 00; 3 Rockwool Foundation, Copenhagen, Denmark; 4 Section of Biostatistcs, Department of Public Health, University of Copenhagen, Copenhagen, Denmark; 5 Section of Environmental Health, Department of Public Health, University of Copenhagen, Copenhagen, Denmark

**Keywords:** divorce, digital health, medication, mental health, observational study, technology, intervention, physical health, long-term, register data, online

## Abstract

**Background:**

Digital health interventions are increasingly promoted as scalable and cost-effective approaches to support mental health and resilience. Short-term benefits are well documented, but evidence on long-term outcomes (beyond 12 mo) remains scarce, particularly when assessed with objective measures in large cohorts. Most studies to date have focused on small samples, relied on self-reported outcomes, and used follow-up periods of less than a year. This leaves uncertainty about whether early changes are sustained over time and whether they can be observed in objective indicators of health. This gap is particularly relevant for stressful life transitions, where the risk of long-term adverse health outcomes is high. Divorce, a common and stressful transition linked to poorer mental and physical health, thus provides an ideal case for investigating the long-term potential of digital health interventions.

**Objective:**

This study examined the association between SES One, a digital health intervention for Danish divorcees, and mental health medication use, primary care usage, and hospitalizations over a 5-year follow-up period using Danish national health registers.

**Methods:**

Participants (n=1856) from a randomized controlled trial of SES One in Denmark were followed for 5 years after divorce. Outcomes included mental health medication prescriptions (eg, antipsychotics, anxiolytics, hypnotics, sedatives, and antidepressants), primary care usage (eg, billable interactions with general practitioners, specialist practitioners, and psychologists), and hospitalizations. Odds ratios and incidence rate ratios were calculated to compare outcomes between SES One participants and the control group.

**Results:**

Over 5 years, SES One participants did not have significantly lower odds of filling a prescription (odds ratio [OR] 0.836; *P*=.09) but filled 28% fewer prescriptions overall (incidence rate ratio 0.720; *P*=.045), indicating a *reduce-not-remove* effect. No overall differences were observed in primary care usage or hospitalizations. However, participants had 38% (OR 0.624, *P*=.003) and 27% (OR 0.730, *P*=.001) lower odds of visiting primary care in years 2 and 3, respectively, and 32% (OR 0.677, *P*=.046) lower odds of hospitalization in year 4, suggesting possible late-onset effects.

**Conclusions:**

The findings advance the field by showing that a targeted digital health intervention can generate measurable long-term health benefits in a large cohort when evaluated with objective registry data. The results suggest that such interventions may reduce reliance on medication and health care services over time, not by eliminating needs entirely but by reducing them. These patterns can be interpreted as reflecting both legacy and late-onset pathways. Long-term evaluations with objective data are essential to fully capture the durability and timing of digital health intervention effects.

## Introduction

### Digital Health Interventions and the Knowledge Gap on Long-Term Effects

Advances in public health and health care have substantially improved survival and quality of life across populations [1,2]. Life expectancy has increased in most countries, and many acute conditions that were once fatal can now be effectively treated or managed [2,3]. However, new challenges persist. Mental health problems remain highly prevalent [4], and many people struggle to maintain resilience when faced with major life events and stressful transitions, such as bereavement, unemployment, or divorce [5-8]. Addressing these challenges requires approaches that support both short-term and long-term health and well-being.

Digital health interventions are increasingly positioned as a promising response. They offer flexible, scalable, and cost-effective access to evidence-based support and have been shown to reduce adverse health outcomes, such as stress [9,10], anxiety [11-13], and depression [11-13], in targeted populations, with evidence also supporting benefits for mental well-being at the population level [14]. Yet, most evaluations are based on short-term trials [15-17], often limited to self-reported outcomes [18,19], challenged by retention and adherence [14,17,20], or conducted with relatively small samples (fewer than 200 participants) [21,22].

This has left uncertainty about whether observed effects of digital health interventions persist over time and translate into measurable changes in more objective health outcomes, such as medical prescriptions, primary care usage, or hospitalizations [14,15,18-20]. Consequently, a key gap remains in understanding whether digital health interventions produce long-term health benefits beyond 12 months, particularly when assessed with measures not affected by self-report, attrition, or insufficient statistical power.

### Divorce in Denmark as an Ideal Case

To address this gap, we use divorce in Denmark as a case. Divorce is a common life transition in Western societies, with divorce rates ranging from 35% to 50% [9,23]. It is consistently linked to worse health outcomes, including higher risks of chronic disease [24,25], depression [26,27], anxiety [27,28], social isolation [24,29], and reduced physical activity [28,30]. Divorced individuals are also reported to have a 30% higher mortality hazard [8] than their married counterparts, a statistic comparable to other major public health risks, such as physical inactivity and obesity [29]. To examine whether digital health interventions can provide long-term support against these risks, Denmark offers a particularly strong setting: digital literacy is high, uptake of online programs is widespread, and comprehensive national registers enable entire trial cohorts to be followed over time with objective outcome measures [31-33]. These features make divorce in Denmark an ideal case for evaluating the long-term health outcomes of digital health interventions.

### Objectives and Hypotheses

The objective of this study is to examine the association between long-term health outcomes and SES One, a digital health intervention developed to support recently divorced individuals in Denmark [9]. SES One is a digital platform accessible online by computer or mobile devices, consisting of learning modules that integrate cognitive behavioral therapy (CBT), narrative therapy, and acceptance and commitment therapy. It was evaluated in a randomized controlled trial (RCT) involving 1856 participants [9]. The RCT demonstrated significant short-term improvements, including reductions in stress, anxiety, depression, and sick days during the first year after divorce [9,34]. Detailed information on the design, methods, analyses, and results of the original RCT can be found in studies by Hald et al [9], Sander et al [34], and Agerholm et al [35].

Building on these findings and on the documented benefits of digital health interventions more broadly, we examine whether the use of SES One is associated with differences in long-term health outcomes. To capture this, we use health care use (including medication use, health care visits, and hospitalizations) as a proxy for health. Health care use is widely applied in longitudinal research, and while short-term help-seeking may support recovery, frequent and persistent use over time has been shown to be associated with prolonged distress and deteriorating physical or mental health [35-39].

We thus link the trial cohort to nationwide registers and follow participants for 5 years. On the basis of the RCT results and existing research in the field, we hypothesize that, compared to controls, participants who had access to SES One will, 5 years after the trial, show (1) reduced mental health medication use (including psycholeptics and antidepressants), (2) less primary care usage (including general practitioner [GP], specialist, and psychologist), and (3) fewer hospitalizations.

Taken together, these outcome measures provide a multidimensional view of somatic and mental health needs and behaviors, as well as provide information on the extent the trial may also lower the health costs associated with divorce. Furthermore, by relying on administrative records, our study is not affected by any differential attrition of respondents in trial follow-up because all trial respondents still alive and in Denmark are consistently included. To our knowledge, this is among the first studies to evaluate long-term (≥5 y) health outcomes of a digital health intervention using objective measures in a large cohort.

## Methods

### Intervention

SES One (‘SES’ is abbreviated from the Danish ‘Samarbejde Efter Skilsmisse,’ which translates to ‘Cooperation After Divorce’) is a comprehensive, fully digital intervention that comprises 17 modules accessible online via computer or mobile devices, such as smartphones or tablets, and is designed to address key challenges faced by divorced parents. The content is grouped into three main themes that broadly align with evidence-based divorce education priorities identified in the literature: the divorcee, the children, and co-parenting [34]. Each module takes approximately 30 to 60 minutes to complete and incorporates interactive exercises, SMART (specific, measurable, attainable, relevant, and time-bound) goals, and multimedia features, such as voiceovers, animations, expert videos, and dramatized scenarios. The intervention integrates elements from multiple psychological approaches, including CBT, narrative therapy, and acceptance and commitment therapy, and was developed as a “born digital” program specifically designed to leverage digital engagement strategies from the outset. Aside from self-reported survey data, the only additional information collected during the RCT was the number of modules completed. SES One functioned purely as a software-based intervention, without access to health professionals or social workers. Participants could access any module at any time and in any order. During the 12-month RCT period, participants with access to SES One completed an average of 4.27 (SD 2.94) modules [9].

### Study Design and Participants

In this retrospective observational study, we observe a treatment group and a control group 5 years after an RCT of SES One. The RCT included 1856 recently divorced Danes who completed an online questionnaire, on average, within a week of their juridical divorce. Study participants were recruited through email invitations distributed by the Danish State Administration, the authority responsible for granting divorce decrees in Denmark [40]. After the online questionnaire, participants were randomly assigned to either the treatment group (n=1031, 55.5%), receiving unlimited access to SES One for 12 months, or the control group (n=825, 44.5%), which did not have access to SES One at any time. The data collection period for the RCT lasted 2 years, from January 2016 to January 2018, with participants invited from January 1, 2016, to January 31, 2017 [9]. For full information and details on the design, methods, analyses, and results of the RCT, please see studies by Hald et al [9], Sander et al [34], and Cipric et al [40].

To conduct the retrospective observational study, we used available Danish register-based data. We linked the deidentified social security numbers of participants in the RCT with Danish national register-based data on the variables of study interest, 5 years after treatment or nontreatment with SES One. As all outcomes were obtained from administrative records, the study did not suffer from bias due to selective attrition in the follow-up period. Only sources of attrition were death and outmigration, which is why any missing information on outcomes was treated as missing at random.

Participants who joined the RCT in early January 2018 but were invited in late 2017 had their treatment date assigned as December 31, 2017 (n=6). This was done to ensure full information on the 5-year follow-up period, which otherwise would not have been possible for this group due to the yearly measurement of primary care usage. We followed STROBE (Strengthening the Reporting of Observational Studies in Epidemiology) reporting guidelines. For the preregistration of this study, please see Hald et al [41].

### Procedures and Variables

Statistics Denmark and the ROCKWOOL Foundation have compiled and organized the data. We submitted requests for the specific data of interest and, upon obtaining it, merged the various datasets using social security identifiers (Central Person Register numbers). The requests, organization, and data administration occurred from December 2023 to August 2024, and the final dataset was ready by September 2024. No data linkage of RCT and administrative data occurred before finalizing preregistration on March 15, 2024.

Data on the participants’ social security numbers, treatment status, marriage duration (in months), conflict level, and time of treatment were sourced from the RCT. Data on background variables, including legal gender (man or woman), age (in years), income (total income from all sources before tax in DKK), educational level (highest completed), migration background (Danish or non-Danish origin), and date of juridical divorce, were sourced from Statistics Denmark’s Population Register, Income Register, and Labor Market Register were measured at baseline (juridical divorce).

Data on the outcome variables were sourced from the Danish Patient Register [33], the Danish National Health Service Register [42], and the Medical Prescription Database [43].

The Medical Prescription Database includes all filled outpatient prescriptions in Denmark, allowing us to generate a measure of the total number of filled prescriptions from the date of juridical divorce until 5 years later outside hospital settings, based on Anatomical Therapeutic Chemical (ATC) codes N05 ‘psycholeptics’ (ATC N05: antipsychotics, anxiolytics, hypnotics, and sedatives) and N06A ‘antidepressants’ and N06C *psycholeptics and psychoanaleptics in combination*. In Denmark, prescriptions of class N06C are not used, so no count of N06C was included in the analyses. Prescriptions are captured at the daily level, allowing us to track individuals from their day of divorce.

Primary care usage represents the total number of billed consultations with general practitioners, specialist practitioners, and publicly funded psychologists from 1 year after the juridical divorce until 5 years later. The data are only available annually and do not report diagnoses nor provide information on date, content, or length of visits. One visit may include more than one billed consultation.

Hospitalizations are measured as a binary variable (yes or no), indicating whether an individual was hospitalized as an inpatient during the period from the date of juridical divorce until 5 years later. The data cover all patients discharged from Danish hospitals. Hospital stays are captured at the daily level, allowing us to track individuals from their day of divorce.

The background and outcome variables have been assessed for all participants included in the study. Descriptive statistics for each background and outcome variable are provided in Table 1, separately for the control group and the treatment group.

**Table 1.** Descriptive statistics for the sample by treatment group (control=0, SES One=1; N=1856).

Variable	Control group (n=825, 44.5%)	Treatment group (n=1031, 55.5%)	Total
New partner, n (%)			
Both	40 (4.8)	48 (4.7)	88 (4.7)
None	533 (64.6)	655 (63.5)	1188 (64.0)
Respondent	83 (10.1)	108 (10.5)	191 (10.3)
For spouse	169 (20.5)	220 (21.3)	389 (21.0)
Gender, n (%)			
Male	262 (32.1)	343 (33.6)	605 (32.9)
Female	555 (67.9)	679 (66.4)	1234 (67.1)
Danish origin^a^, n (%)			
No	63 (7.6)	96 (9.3)	159 (8.6)
Yes	762 (92.4)	935 (90.7)	1697 (91.4)
Educational attainment, n (%)			
Short (ISCED^b^ 0‐2)	60 (7.3)	74 (7.2)	134 (7.2)
Medium (ISCED 3‐4)	257 (31.2)	307 (29.8)	564 (30.4)
Long (ISCED 5+)	508 (61.6)	650 (63.0)	1158 (62.4)
Divorce month, n (%)			
January	66 (8.0)	109 (10.6)	175 (9.4)
February	68 (8.2)	86 (8.3)	154 (8.3)
March	83 (10.1)	100 (9.7)	183 (9.9)
April	61 (7.4)	83 (8.1)	144 (7.8)
May	69 (8.4)	80 (7.8)	149 (8.0)
June	52 (6.3)	87 (8.4)	139 (7.5)
July	80 (9.7)	87 (8.4)	167 (9.0)
August	73 (8.8)	98 (9.5)	171 (9.2)
September	69 (8.4)	74 (7.2)	143 (7.7)
October	70 (8.5)	86 (8.3)	156 (8.4)
November	70 (8.5)	79 (7.7)	149 (8.0)
December	64 (7.8)	62 (6.0)	126 (6.8)
Divorce year, n (%)			
2015	43 (5.2)	47 (4.6)	90 (4.8)
2016	405 (49.1)	557 (54.0)	962 (51.8)
2017	377 (45.7)	427 (41.4)	804 (43.3)
Conflict Scale (5-26)^c^, mean (SD)	13.695 (4.845)	13.787 (4.988)	13.746 (4.924)
Age, mean (SD)	45.187 (8.533)	45.126 (8.545)	45.153 (8.537)
Duration of marriage^d^, mean (SD)	12.627 (8.073)	12.827 (7.993)	12.738 (8.027)
Income (in DKK)^e^, mean (SD)	476,311 (386,284)	484,044 (283,122)	480,607 (332,861)

a Danish origin entails at least one parent of the respondent had Danish citizenship.

b ISCED: International Standard Classification of Education.

c n=1795 (n_treatment_=998; n_control_=797).

d n=1839 (n_treatment_=1022; n_control_=817).

e n=1852 (n_treatment_=1029; n_control_=823).

### Statistical Methods

Stata/MP 18.0 (Stata Corp) and R 4.5.1 (R Development Core Team) were used for the analyses. The sample consisted of 1856 participants (treatment group, n=1031, 55.5%; and control group, n=825, 44.5%), with the only source of attrition being natural due to outmigration or death (as we expand on this in the Results section). As per preregistration, inferences were made using 2-tailed tests, with *P* values less than .05 considered statistically significant. For the by-year-since-divorce results, we estimated separate models by year.

To compare the treatment group and the control group on the count variables (medication prescription index and primary care usage index), we used an uncontrolled negative binomial regression model for each outcome. The results are presented as incidence rate ratios (IRRs) interpreted as ratios of expected counts with associated CIs and *P* values. Moreover, because the primary care usage index is measured annually, we also conducted a sensitivity analysis to examine the effect of starting the count in the year of divorce. These results are provided in the supplementary materials in Multimedia Appendix 1. Finally, especially the medical prescription index has a high share of respondents with a value of zero (n=1342, 72%), we also provide results from a binary logistic regression of any prescriptions filled. Less than 1% of respondents (n=18) had no primary care usage, so we do not include binary regression results for this variable.

To compare the treatment and control groups on hospitalization, we used an uncontrolled logistic regression. We opted for logistic regression over negative binomial regression due to an expected high number of zeros, which was confirmed with 77% of values being zero 5 years after juridical divorce. Results are presented as odds ratios [ORs] with corresponding CIs and *P* values.

### Ethical Considerations

#### Ethics Review Approvals or Exemptions

This 5-year, posttrial observational analysis used pseudo-anonymized Danish administrative registers linked to the randomized trial cohort within Statistics Denmark’s secure environment. Under national regulations governing research use of Statistics Denmark data and in accordance with the General Data Protection Regulation, this secondary analysis did not require additional institutional review board or research ethics board approval. For the original RCT that generated the cohort, procedures complied with the Declaration of Helsinki; the study was approved by the Danish Data Protection Agency and deemed exempt from further ethical evaluation by the Scientific Ethical Committees of Denmark [9].

#### Informed Consent

For the original RCT, participants were informed about the study and provided informed consent before enrollment and randomization [9]. The present register-based follow-up involved a secondary analysis of deidentified administrative data; because individual identities were not available to the research team and analyses were conducted under Statistics Denmark’s legal framework, additional consent and recontact were not required.

#### Privacy and Confidentiality

For the original RCT, survey responses were anonymized and stored on secure servers. For this study, linkage used deidentified Central Person Register keys within Statistics Denmark; data were analyzed on servers hosted at Statistics Denmark and cannot be transferred outside this environment. Outputs were limited to aggregated, disclosure-controlled statistics, and all processing complied with the General Data Protection Regulation.

#### Compensation

No compensation was provided to participants, either for participation in the original RCT or for this secondary register-based follow-up.

#### Identifiable Images

The manuscript and its supplementary material contain no images of individual participants; only aggregate, nonidentifiable figures are presented. Accordingly, no image-based consent was required.

## Results

A total of 1856 participants were recruited for the original SES One trial, and all were included in the 5-year follow-up. During this period, 3.1% (n=58) of respondents exited the study due to either migration or death. While this attrition was slightly higher in the treatment group (n=33, 3.2%) compared to the control group (n=25, 3.0%), the difference was small and not statistically significant (0.2% difference, 95% CI: −1.4% to 1.8%). As attrition appeared independent of treatment status, all respondents were included in all analyses.

Table 1 presents the baseline characteristics of the respondents. The majority were women (n=1234, 67.1%), and the average age was 45.2 years. Additionally, 62.4% (n=1158) of respondents had a degree above high school, the average income was DKK 480,607 (US $75,353), and 91.4% (n=1697) of respondents were born to at least one parent with Danish citizenship. The average marriage duration was 12.8 years. The characteristics were balanced across treatment groups.

Table 2 presents the effects of SES One participation on filled medication prescriptions, primary care usage, and hospitalizations over the 5-year observation period. SES One participants had a 28.0% lower expected count of filled prescriptions compared to the control group (IRR 0.720, 95% CI 0.522-0.993; *P=*.045), which, based on the model, corresponded to 1.34 fewer filled prescriptions in the treatment group (4.77 for the control group and 3.43 for the treatment group) and a 1.9 percentage-point higher probability of not filling any prescription (71.1% for the control group and 73.1% for the treatment group, a nonsignificant difference).

**Table 2.** Comparison of the number of prescriptions and primary care usage and the odds of hospitalization between treatment groups (control=0 and SES One=1; N*=*1856).

Variable	IRR^a^/OR^b^ (95% CI)^c^		*P* value
Medical prescription index	IRR 0.720 (0.522-0.993)		.045
Primary care usage index	IRR 0.944 (0.869-1.025)		.17
Hospitalization	OR 0.851 (0.685-1.058)		.15

a IRR: incident rate ratio.

b OR: odds ratio.

c See Table S1 in Multimedia Appendix 1 for log IRR and log OR.

For primary care usage, SES One participants had a 5.6% lower expected count than the control group (IRR 0.944, 95% CI 0.869-1.025; *P=*.17) corresponding to 2.89 fewer visits (51.54 for the control group and 48.64 for the treatment group) and <0.1 percentage-point difference in the probability of zero visits; neither of these estimates reached statistical significance. Regarding hospitalization risk, SES One participants had 14.9% lower odds of spending any night in the hospital compared to the control group (OR 0.851, 95% CI 0.685-1.058; *P=*.15) corresponding to a 2.9 percentage-point difference (24.5% in the control group and 21.6% in the treatment group), but this result also did not reach statistical significance.

**Figure 1.**
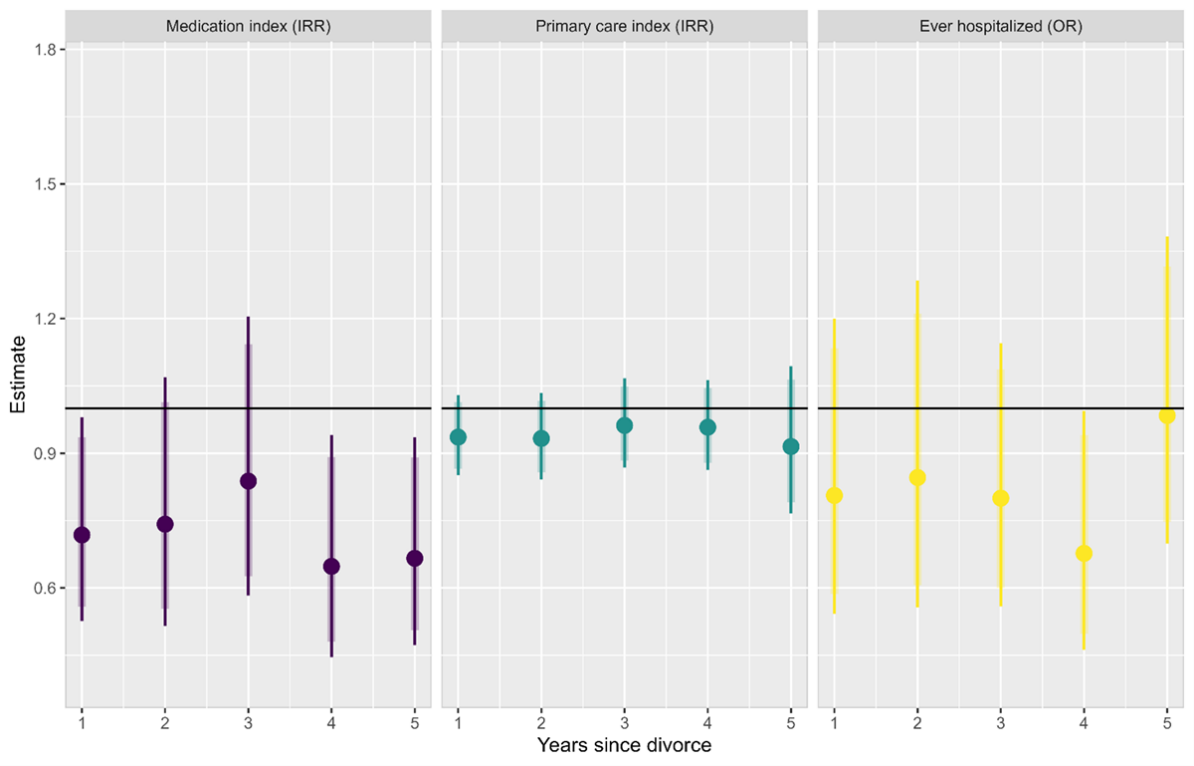
Year-by-year comparison of the number of prescriptions and primary care usage and the odds of hospitalization between treatment groups. Thin lines represent 95% CI. Thick lines represent 90% CI. See Table S2 in Multimedia Appendix 1 for numerical estimates. IRR: incident rate ratio; OR: odds ratio.

Figure 1 presents the effects of SES One participation on medication prescriptions, primary care usage, and hospitalizations for each year following divorce. For the medication prescriptions, the annual IRRs are all below one, ranging from 0.648 (95% CI 0.446-0.941; *P=*.02) to 0.838 (95% CI 0.583-1.205; *P=*.34) with SES One participants having statistically significant lower expected counts than the control group in years 1, 4, and 5 after juridical divorce. For the primary care usage index, IRR estimates range from 0.915 in year 5 (95% CI 0.766-1.094; *P=*.33) to 0.962 in year 3 (95% CI 0.868-1.067; *P=*.47), with no estimates reaching statistical significance. Finally, for hospitalizations, ORs range from 0.677 in year 4 (95% CI 0.462-0.994; *P=*.046) to 0.984 in year 5 (95% CI 0.699-1.383; *P=*.93) with SES One participants having statistically significant lower odds of hospitalization compared to the control group in the fourth year after divorce.

Table 3 and Figure S1 in Multimedia Appendix 1 show SES One’s effects on filling at least one medical prescription and having a primary care usage over 5 years and by individual year postdivorce. Over 5 years, SES One participants had lower, though not statistically significant, odds of filling a prescription (OR 0.836, 95% CI 0.681-1.025; *P*=.09) or having primary care usage (OR 0.477, 95% CI 0.169-1.345; *P*=.16). However, in years 4 (OR 0.770, 95% CI 0.595-0.997; *P*=.048) and 5 (OR 0.770, 95% CI 0.595-0.995; *P*=.046), SES One participants had significantly lower odds of filling a prescription. They also had significantly lower odds of primary care usage in years 2 (OR 0.624, 95% CI 0.457-0.851; *P*=.003) and 3 (OR 0.730, 95% CI 0.542-0.983; *P*=.04). Over time, SES One participants show a slight trend toward fewer prescriptions, while their primary care usage odds gradually align with the control group’s, largely driven by GP contacts. Supplementary Figure S2 in Multimedia Appendix 1 and Tables S5 and S6 in Multimedia Appendix 1 confirm these findings, with additional details on visit types (GP, psychologist, and other specialists).

**Table 3.** Comparison of the odds of filling any prescription and having any primary care usage between the treatment groups (control=0 and SES One=1; N=1856).

Variable	OR^a^^,^^b^ (95% CI)		*P* value
Medical prescription index	0.836 (0.681-1.025)		.09
Primary care usage index	0.477 (0.169-1.345)		.16

a OR: odds ratio.

b Table S5 in Multimedia Appendix 1 provides log OR.

We examine heterogeneity in the medication and primary care usage indices. The medication index includes psycholeptics (ATC N05: antipsychotics, anxiolytics, and hypnotics or sedatives) and antidepressants (ATC N06A). Table 4 shows that SES One participants had a 41% lower expected count of psycholeptic prescriptions (antipsychotics, anxiolytics, hypnotics, and sedatives) over 5 years (IRR 0.588, 95% CI 0.363-0.950; *P*=.03; treatment group average 1.29 vs 2.19 for the control group) and a nonsignificant 17% lower count for antidepressants (IRR 0.833, 95% CI 0.618-1.112; *P*=.23; treatment group average 2.15 vs 2.58 for the control group). Figure S3 in Multimedia Appendix 1 shows that SES One participants had significantly lower psycholeptics counts in years 1, 2, 4, and 5 postdivorce.

**Table 4.** Comparison of the number of psycholeptics and antidepressants prescriptions between treatment groups (control=0 and SES One=1; N=1856).

Variable	IRR^a^ (95% CI)		*P* value
Psycholeptics	0.588 (0.363-0.950)		.03
Antidepressants	0.833 (0.618-1.122)		.23

a IRR: incident rate ratio.

b See Table S5 in Multimedia Appendix 1 for log IRR.

### Expanding Results Beyond the Trial Population

The SES trial participants were recruited from the full universe of Danish divorcees during the trial period, but do not reflect the average divorcee in the period. Previous work has shown that participants were more likely to be women, experiencing their first divorce, and holding longer education [9]. To expand the results beyond the trial participants, we reweight the key findings from the trials to the target population of all Danish residents who finalized a divorce in the same period as the trial took place using calibration weighting [44]. The reweighting is based on a set of covariates that need to be sufficient to capture: “all variables that are related to the trial participation and outcome” [44]. Given that this is a very strong assumption, the results of the calibration weighting should be viewed as providing absolute upper-bound effect estimates under the scenario where, conditional on covariates, the target population would see similar effects of the intervention as the trial population. We limit the results displayed to those found in Tables 2 and 4, and to avoid issues of extreme weights based on few observations, we exclude individuals aged 70 years or higher or who had been married for 45 years or more from both the RCT and the target population (n=57,793).

Table 5 reports the results from the calibration weighting contrasted with the results from the RCT population reported in Tables 2 and 4. Although results are weighted across eight covariates, they differ little from the results reported from the RCT, with the exception of the primary care index, for which the calibration weighted results suggest that an 11.9% decrease in primary care usage (IRR 0.881, 95% CI 0.783-0.996; *P*=.04; and treatment group average of 45.22 vs 51.30 for the control group) compared to the 5.6% decrease for the RCT sample. Effects on filled prescriptions for psycholeptics were also significant at the 5% level (IRR 0.616, 95% CI 0.395-1.0; *P*=.048; and treatment group average of 1.211 vs 1.964 for the control group).

**Table 5.** Calibration weighted estimates to target population of all Danish residents finalizing a divorce during the trial period (n=57,793)^a^.

Variable	RCT^b^ sample	Calibration weighted		
	IRR^c^/OR^d^	IRR/OR (95% CI)		*P* value
Medical prescription index	IRR 0.720	IRR 0.751 (0.516-1.091)		.14
Psycholeptics	IRR 0.588	IRR 0.616 (0.395-1.000)		.048
Antidepressants	IRR 0.833	IRR 0.834 (0.537-1.260)		.40
Primary care usage index	IRR 0.944	IRR 0.881 (0.781-0.995)		.04
Hospitalization	OR 0.851	OR 0.877 (0.655-1.211)		.40

a SEs and 95% CIs obtained from 10,000 bootstrap repetitions. *P* value calculated parametrically with bias-corrected estimate. CIs were obtained nonparametrically. To ensure no extreme weights and model convergence, both randomized controlled trial (RCT) and target sample exclude all people who divorced following a marriage longer than 44 years, at an age of 70 years or higher, or did not have observed information on income year before divorce (26 individuals from the RCT). The following set of covariates measured at baseline is included in the calibration weighting: gender (binary), Danish origin (binary), first divorce (binary), any biological children (binary), age (continuous), marriage length (continuous), education (categorical: lower secondary, upper secondary, tertiary), and income (in DKK 10,000K).

b RCT: randomized controlled trial.

c IRR: incident rate ratio.

d OR: odds ratio.

## Discussion

### Principal Findings

The aim of this study was to address the limited evidence on the long-term health outcomes of digital health interventions. By linking a large RCT cohort of recently divorced individuals in Denmark to nationwide registers and following them for 5 years, we were able to assess objective outcomes related to mental health medication use, health care visits, and hospitalizations. The results showed that SES One participants did not have statistically significant lower odds of filling a prescription (OR 0.836, 95% CI 0.681‐1.025; *P*=.09) but filled 28% fewer prescriptions over the 5-year period (IRR 0.720, 95% CI 0.522-0.993; *P*=.045) compared to controls, suggesting that the digital health intervention reduced, but not fully removed, the need for medication. This pattern was most evident for psycholeptics and became most pronounced in the fourth year after divorce. No overall differences were found in primary care usage or hospitalizations, but fewer GP visits in years 2 and 3 and fewer hospitalizations in year 4 suggest that some benefits may be late onset.

When the RCT results were reweighted to the full universe of divorcees in Denmark from the same period as the trial ran, the results showed that under the assumption that the covariates included in the recalibration fully accounted for selection into trial participation and the outcome (a strong assumption), the effect of the RCT remained largely unchanged. Given the strict assumption underlying the transportability estimation, this should, however, be viewed as upper bounds on the effect.

### Comparison With Prior Work

Systematic reviews show short-term benefits of digital health interventions for depression, anxiety, and well-being, especially when CBT components and human support are included, but these effects are generally modest and heterogeneous [14,15,19,20]. Studies of fully automated interventions find smaller effects and highlight problems with engagement and adherence [17,23]. Some evidence suggests that resilience- and well-being–focused interventions can sustain benefits up to 12 months, but effects are small [16,18]. Earlier studies on SES One and comparable interventions for divorcees also demonstrate short-term moderate to large health benefits, but these findings are likewise constrained by short follow-up periods or small sample sizes [2,3,43-48].

Longer-term evaluations of digital health interventions are therefore rare, with only a few exceptions. A study on internet-based CBT for social anxiety found durable improvements after 5 years, suggesting that skills learned online can persist without continued contact, although the findings were based on self-report and follow-up participation was selective [24]. Another study on app-supported follow-up after cardiac rehabilitation showed short-term gains that had waned by year 5 once structured support ended [25]. Finally, a 5-year study on a web-based behavior change system for obesity did not sustain weight loss, yet participants started antihypertensive medication less often, pointing to a legacy of early risk factor improvement [49].

Taken together, most evaluations of digital health interventions have been limited by small samples, self-reported outcomes, and follow-up periods of less than 12 months. Against this backdrop, this study advances the field by demonstrating long-term associations between a targeted digital health intervention and objective health outcomes in a large cohort. The findings also open new ways of understanding such interventions by suggesting a “reduce-not-remove” pattern of effects and by pointing to both legacy and late-onset pathways in long-term outcomes.

### Reduce-Not-Remove Effect

Interpreting the observed results on mental health prescription use suggests a “reduce-not-remove” effect pattern (ie, dose reduction rather than full discontinuation [45]). The observed difference between the treatment and control groups in the odds of filling a prescription (‘remove’) versus the difference in the expected count of filled prescriptions (‘reduce’) implies that the primary difference between the two groups was a significant and large reduction in the number of prescriptions in the treatment group, not a complete discontinuation of medication. This suggests that the psychosocial tools, strategies, and techniques provided through the intervention helped participants manage stress and emotional distress more effectively [9,28,34], thereby reducing (but not removing) the need for pharmacological treatment. Such partial but meaningful reductions may be a more realistic expectation for the long-term health outcomes of digital health interventions rather than complete discontinuation of treatment.

### Legacy and Late-Onset Effects

The observed time-varying associations between treatment and health outcomes point to two possible pathways of long-term health outcomes. One possible pathway is through legacy effects, where the early psychosocial benefits of SES One in the first year after divorce may have helped stabilize participants’ mental health and equipped them with skills to cope with the risks immediately after the divorce. This legacy effect was then sustained over time, contributing to long-term benefits, as seen, for example, in the continued reduction in medication use. The other possible pathway is through late-onset effects, where health benefits only became visible after several years. This interpretation is supported by the observed reductions in GP visits in years 2 and 3 and in hospitalizations in year 4, despite no initial reductions in year 1. In this interpretation, the intervention’s early impact on mental health may have initiated a longer process in which improved psychosocial stability gradually translated into better health outcomes over time compared with controls. For example, non-SES users may have experienced unaddressed psychological distress that accumulated and eventually deteriorated into physical health problems, whereas SES users were protected from such late deterioration due to early stabilization provided by the digital intervention.

Thus, both legacy and late-onset effects may be at play: legacy effects reflecting the initial psychological benefits gained from the intervention and late-onset effects reflecting protection against delayed deterioration.

### Implications for Practice and Future Research

The findings suggest that digital health interventions such as SES One can provide accessible, nonpharmacological support for individuals navigating major life transitions, such as divorce. By reducing (but not removing) the need for mental health medication and potentially lessening the use of health care services later on, such interventions may complement existing services in primary care and mental health. In practice, this shows the value of integrating digital health into broader care pathways, not as replacements for traditional services, but as scalable tools that can help stabilize well-being and buffer the long-term health risks associated with stressful life events.

Future studies should build on this work by evaluating digital health interventions across different populations, life transitions, and health care contexts to assess generalizability. Longer-term follow-ups using objective measures are essential to understand whether observed benefits are sustained, delayed, or heterogeneous across subgroups. Research should also explore the mechanisms behind legacy and late-onset pathways, including how digital tools interact with health care use, social support, and resilience over time. Finally, comparative studies that include cost-effectiveness analyses could clarify the potential of digital health interventions to complement existing health and welfare systems at scale.

### Limitations

The study also has limitations. First, we could not distinguish between GP visits for mental health versus routine care, which may underestimate SES One’s impact. Second, private psychologist visits and other health services were not tracked, possibly missing some of SES One’s benefits. Third, annual tracking of primary care usage may overlook timing nuances, and measuring only from the posttreatment year may have missed initial effects, while including the treatment year risked pretreatment bias. Fourth, the COVID-19 pandemic, which overlapped with part of the study, could have influenced health care usage, adding or limiting between-group variability. Specifically, individuals may have changed behavior surrounding primary health care usage, which could drive estimates toward zero. Finally, generalizability to other countries may be limited by Denmark’s context, where universal health care and welfare support may have diluted effects [46,47], whereas high digital literacy and advanced digital infrastructure likely strengthened engagement [31-33].

### Conclusions

This 5-year follow-up of a postdivorce RCT cohort shows that a digital health intervention was linked to improved long-term health outcomes. The intervention was associated with reduced use of mental health medication over the 5-year period, with reductions in GP visits and hospitalizations observed in specific years. These findings were interpreted as a “reduce-not-remove” pattern and pointed to both legacy and late-onset effects. When findings were transported to the population of all Danish divorces during the trial period using calibration weighting, results remained largely unchanged. Together, the findings indicate that digital health interventions may provide long-term, nonpharmacological support during major life transitions and demonstrate the importance of extended follow-up of digital health interventions, as long-term health outcomes may vary in timing and intensity.

## Supplementary material

10.2196/69387 Multimedia Appendix 1 Supplementary and supporting tables and figures.

## Abbreviations

ATC: Anatomical Therapeutic Chemical
CBT: cognitive behavioral therapy
CPR: Central Person Register (Danish social security number system)
GP: general practitioner
IRR: incidence rate ratio
OR: odds ratio
RCT: randomized controlled trial
SES: Samarbejde Efter Skilsmisse
SMART: specific, measurable, attainable, relevant, and time-bound
STROBE: Strengthening the Reporting of Observational Studies in Epidemiology
