# Supplementary material for: Digital Health Intervention for and Long-Term Health Outcomes of a Divorce Cohort With Linked Danish Data: 5-Year Posttrial Follow-Up of a Randomized Controlled Trial
Source: J Med Internet Res. 2025 Dec 30;27:e69387. doi: 10.2196/69387 (PMC12753028; doi:10.2196/69387)
Supplement: Multimedia Appendix 1 [file jmir-v27-e69387-s001.docx]

| Table S1: Results from regression of main outcomes on treatment status as log incidence rate ratios and log odds ratios | | | |
| --- | --- | --- | --- |
|  | Medical prescription index  log(IRR) | Primary care visit index log(IRR) | Hospitalization log(OR) |
| SES one vs. control | -0.328^*^ | -0.058 | -0.161 |
|  | [-0.650,0.007] | [-0.140,0.024] | [-0.378,0.056] |
|  | *P*=.045 | *P*=.167 | *P*=.146 |
| *N* | 1856 | 1856 | 1856 |

IRR: Incident Rate Ratio. OR: Odds Ratio.

95% confidence intervals in brackets.

^+^ *P*< 0.1, ^*^ *P*< 0.05, ^**^ *P*< 0.01

| Table S2: Estimates from Figure 1. | | | | | |
| --- | --- | --- | --- | --- | --- |
|  | Year 1 | Year 2 | Year 3 | Year 4 | Year 5 |
| SSRI (IRR) | 0.718^*^ | 0.742 | 0.838 | 0.648 | 0.666 |
|  | [0.526,0.980] | [0.515,1.069] | [0.583,1.205] | [0.446,0.941] | [0.473,0.936] |
|  | *P*=.037 | *P*=.109 | *P*=.339 | *P*=.023 | *P*=.019 |
| Primary care index (IRR) | 0.936 | 0.933 | 0.962 | 0.958 | 0.915 |
|  | [0.851,1.030] | [0.842,1.034] | [0.868,1.067] | [0.863,1.063] | [0.766,1.094] |
|  | *P*=.082 | *P*=.174 | *P*=.187 | *P*=.416 | *P*=.332 |
| Hospitalization (OR) | 0.806 | 0.846 | 0.800 | 0.677^*^ | 0.984 |
|  | [0.542,1.200] | [0.557,1.285] | [0.559,1.145] | [0.462,0.994] | [0.699,1.383] |
|  | *P*=.288 | *P*=.434 | *P*=.222 | *P*=.046 | *P*=.924 |
| *N* | 1856 | 1856 | 1856 | 1856 | 1856 |

IRR: Incident Rate Ratio. OR: Odds Ratio.

95% confidence intervals in brackets.

| Table S3: Results from regression of main outcomes coded as binary indicator on treatment status as log odds ratios | | | |
| --- | --- | --- | --- |
|  | Medical prescription index  log(OR) | Primary care visit index log(OR) |  |
| SES one vs. control | -0.179^+^ | -0.739 |  |
|  | [0.384,0.025] | [-1.775,0.297] |  |
|  | *P*=.085 | *P*=.162 |  |
| *N* | 1856 | 1856 |  |

OR: Odds Ratio.

95% confidence intervals in brackets.

| Table S4: Estimates from Figure 2 | | | | | |
| --- | --- | --- | --- | --- | --- |
|  | Year 1 | Year 2 | Year 3 | Year 4 | Year 5 |
| Primary care (OR) | 0.881 | 0.624 | 0.730 | 0.855 | 0.895 |
|  | [0.651,1.193] | [0.457,0.851] | [0.542,0.983] | [0.669,1.094] | [0.742,1.080] |
|  | *P*=.413 | *P*=.003 | *P*=.038 | *P*=.213 | *P*=.248 |
| SSRI (OR) | 0.831 | 0.801 | 0.800 | 0.770 | 0.770 |
|  | [0.656,1.053] | [0.622,1.031] | [0.622,1.030] | [0.595,0.997] | [0.595,0.995] |
|  | *P*=.125 | *P*=.085 | *P*=.083 | *P*=.048 | *P*=.046 |
| *N* | 1856 | 1856 | 1856 | 1856 | 1856 |

95% confidence intervals in brackets

| Table S5: Results for number of visits using primary care index and decomposed into types of care from year of divorce and followed until four year after divorce | | | | |
| --- | --- | --- | --- | --- |
|  | GP visit  (IRR) | Psychologist visits (IRR) | Specialist visits  (IRR) | Primary care index (IRR) |
| Treatment | 0.934 | 0.958 | 0.982 | 0.942 |
|  | [0.862,1.012] | [0.734,1.250] | [0.842,1.145] | [0.871,1.018] |
|  | *P*=.095 | *P*=.752 | *P*=.815 | *P*=.129 |
| N | 1856 | 1856 | 1856 | 1856 |

IRR: Incident Risk Ratio.

Exponentiated coefficients; 95% confidence intervals in brackets.

| Table S6: Results for number of visits using primary care index and decomposed into types of care from year of divorce and followed until four year after divorce | | | | |
| --- | --- | --- | --- | --- |
|  | GP visit  (OR) | Psychologist visits (OR) | Specialist visits  (OR) | Primary care index (OR) |
| Treatment | 0.249 | 1.069 | 0.878 | 0.249 |
|  | [0.029,2.137] | [0.843,1.355] | [0.725,1.063] | [0.029,2.137] |
|  | *P*=.205 | *P*=.581 | *P*=.182 | *P*=.205 |
| N | 1856 | 1856 | 1856 | 1856 |

OR: Odds Ratio.

Exponentiated coefficients; 95% confidence intervals in brackets.

| Table S7: Results from regression of medical prescription subtypes on treatment status as log risk ratios | | | |
| --- | --- | --- | --- |
|  | Psycholeptics log(IRR) | Antidepressants log(IRR) |  |
| Treatment | -0.531 | -0.183 |  |
|  | [-1.013,-0.051] | [-0.482,0.115] |  |
|  | *P*=.030 | *P*=.229 |  |
| *N* | 1856 | 1856 |  |

IRR: Incident Risk Ratio.

95% confidence intervals in brackets.

| Figure S1: Year by year comparison of the odds of filling any prescription and having any primary care visit between the treatment groups |
| --- |
| 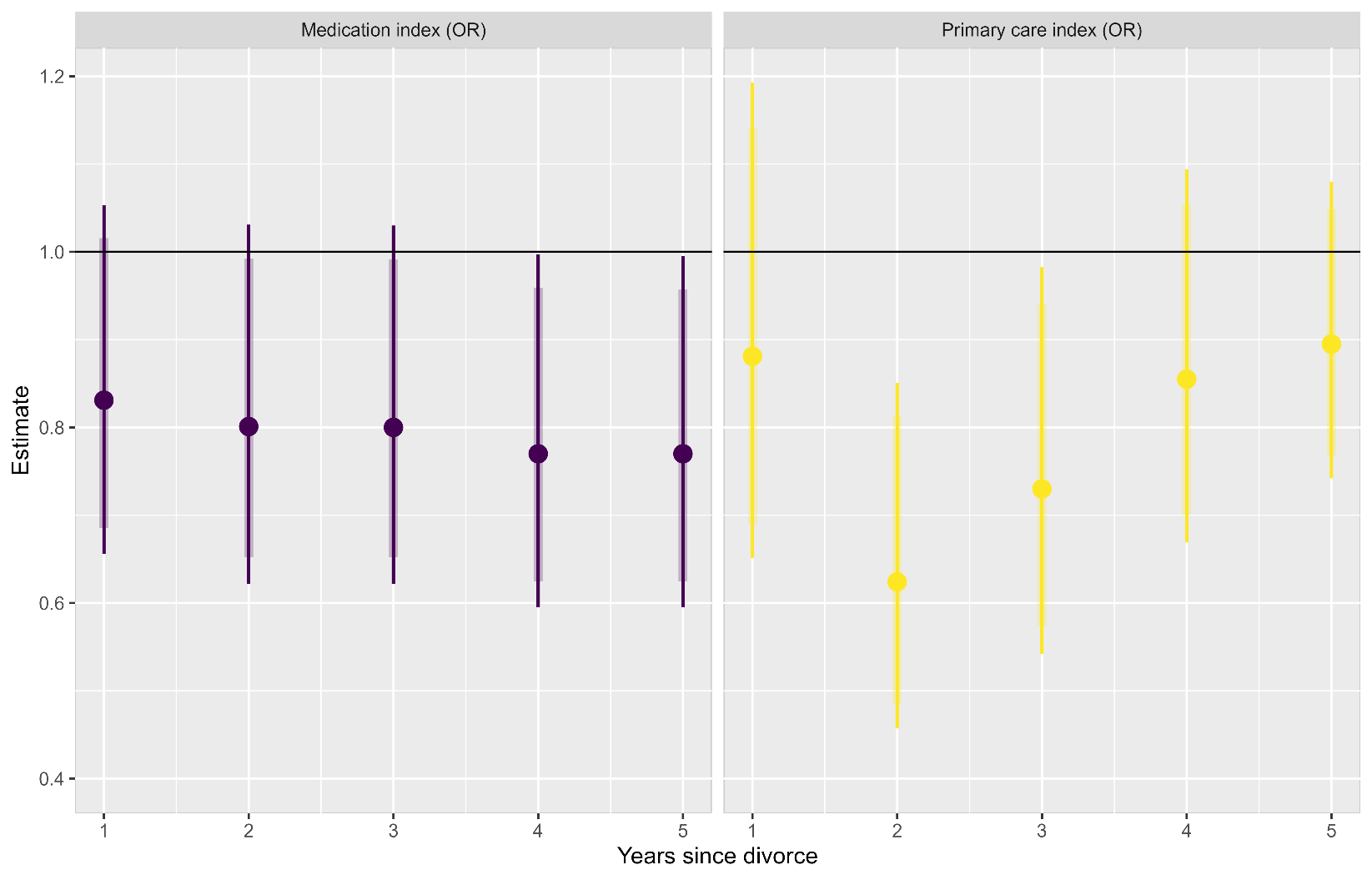 |
| OR: Odds Ratio. Thin lines represent 95% confidence interval. Thick lines represent 90% confidence interval. See Table S4 for numerical estimates. |

Figure S2: By year estimates of decomposed primary care index outcomes on treatment status as both count and binary indicators.
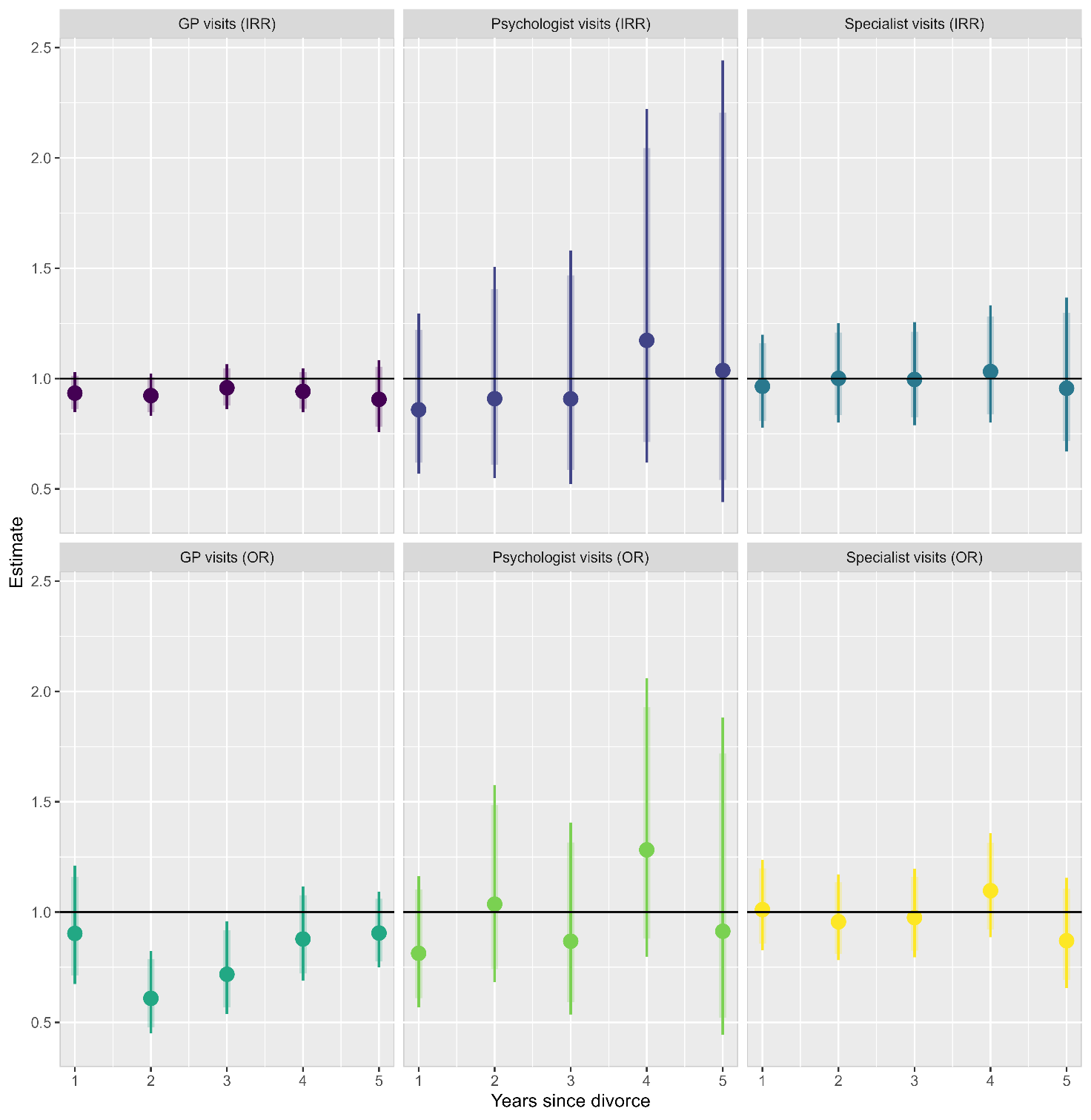

IRR: Incident Risk Ratio. OR: Odds Ratio. Thin lines represent 95% confidence interval. Thick lines represent 90% confidence interval.

Figure S3: By year estimates of medication subcategories outcomes on treatment status.


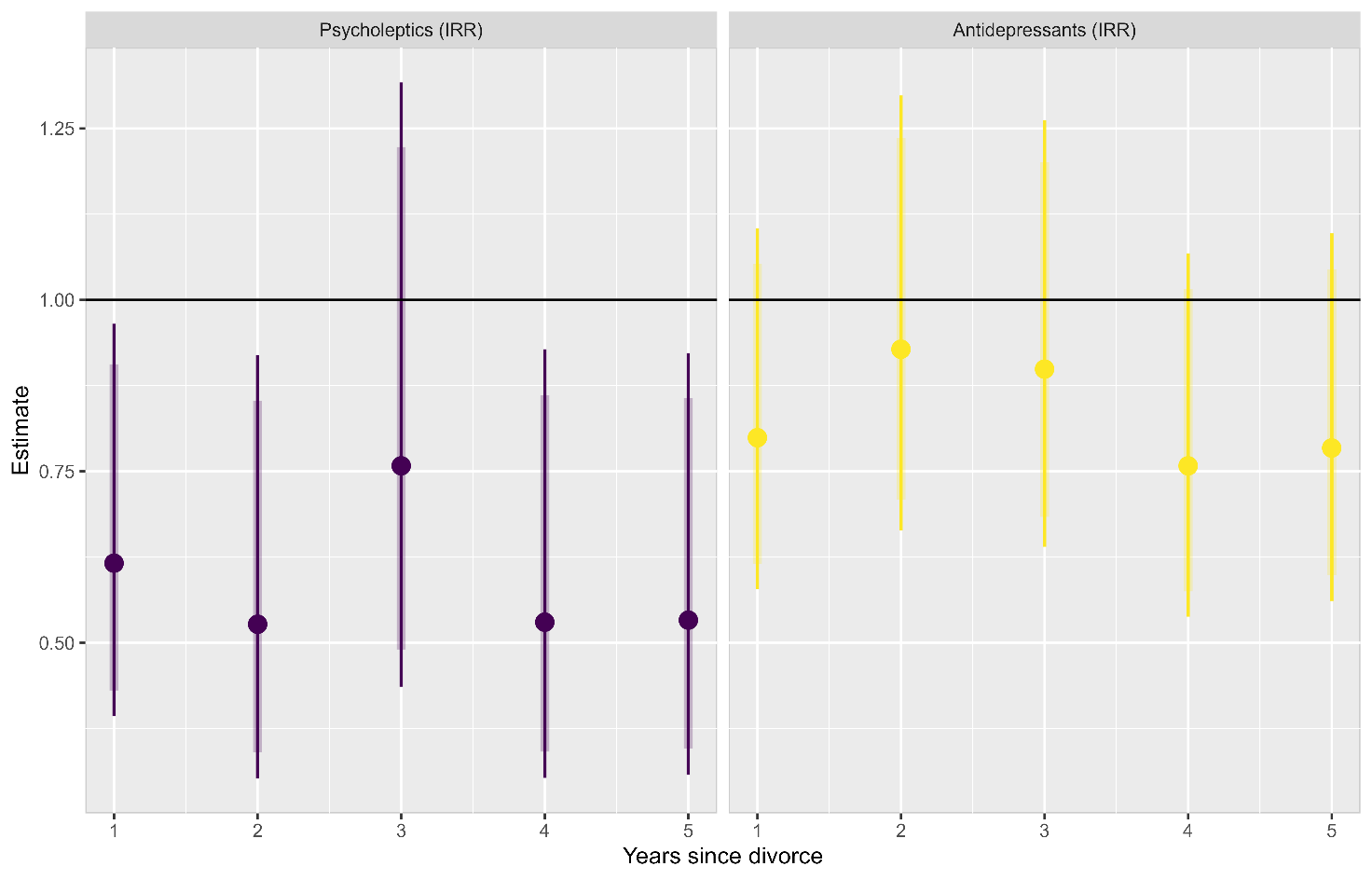

IRR: Incident Rate Ratio. Thin lines represent 95% confidence interval. Thick lines represent 90% confidence interval.
